# Topical ruxolitinib in the treatment of refractory facial seborrheic dermatitis

**DOI:** 10.1016/j.jdcr.2022.04.003

**Published:** 2022-04-23

**Authors:** Eleanor Pope, Eric Kowalski, Francisco Tausk

**Affiliations:** Department of Dermatology, University of Rochester Medical Center, Rochester, New York

**Keywords:** JAK inhibitor, rosacea, seborrheic dermatitis, topical ruxolitinib, JAK, Janus kinase, SD, seborrheic dermatitis

## Introduction

Seborrheic dermatitis (SD) is a chronic, inflammatory skin condition that affects sebum-rich areas of the body.^1^^,^^2^ It is one of the most common skin diseases, with a prevalence of 14.3% in the middle-aged and elderly population.^3^ SD follows a relapsing and remitting course, worsening with stress and during the winter months.^1^ Importantly, SD has been reported to have a negative effect on quality of life.^2^ The underlying mechanism of SD is poorly understood; a combination of *Malassezia* species colonization, immune system activation, and genetic predisposition, among various other endogenous and exogenous factors, likely contribute to its pathogenesis.^4^ The clinical presentation ranges from simple dandruff to a fulminant rash.^4^ The diagnosis is made clinically by the presence of greasy yellow scales overlying well-demarcated erythematous patches or plaques affecting the face, scalp, and upper portion of the chest in a symmetric distribution; the hairline, eyebrows, glabella, and nasolabial folds are particularly involved. Facial SD is frequently associated with rosacea. The goal of therapy is to clear visible signs of disease and reduce associated symptoms and must be maintained long-term to prevent recurrence.^2^ Since the underlying mechanism involves, at least in part, *Malassezia* proliferation and inflammation, common treatments include antifungal and anti-inflammatory therapy.^5^ First-line therapy includes topical antifungals (eg, ketoconazole 2% cream) in combination with a mild topical corticosteroid (eg, hydrocortisone 2.5% or desonide 0.05% creams) or topical calcineurin inhibitor (eg, tacrolimus 0.1% ointment).^1^ With concomitant rosacea, metronidazole 1% gel or cream has been reported to help both conditions.^6^

## Case report

**Fig 1:**
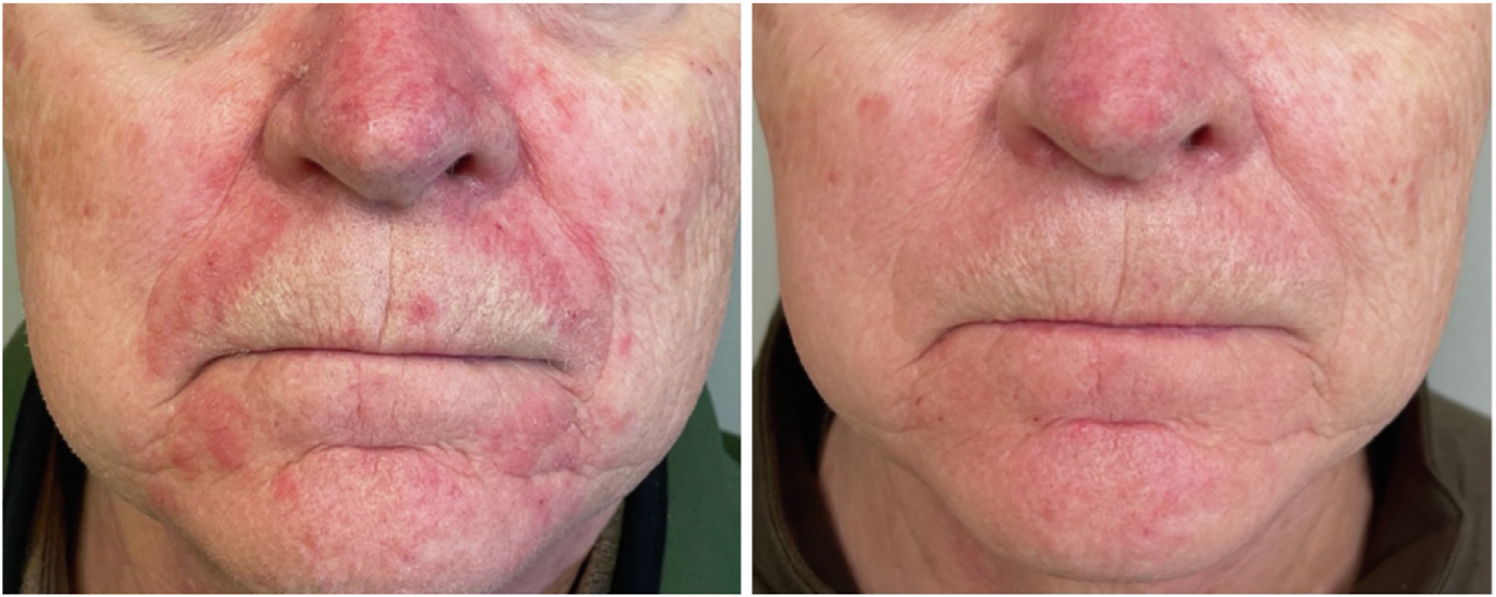
Facial seborrheic dermatitis before (*left*) and after (*right*) 2 weeks of treatment with ruxolitinib 1.5% cream.

Here, we present the case of a 74-year-old man with an unremarkable medical history, who presented to our clinic with a chief complaint of redness and scaling on the nasolabial folds. He had no relief of his symptoms in the past with topical ketoconazole, desonide, and hydrocortisone creams. On examination, there were several red plaques with overlying greasy scale over the nasolabial folds and periorally, without pustules or papules (Fig 1). Additionally, we could appreciate the presence of significant erythema and telangiectasias localized in the central face, for which he had been prescribed metronidazole 1% gel and doxycycline 100 mg daily without improvement. Clinically, we made a diagnosis of rosacea, with a marked presence of SD, for which we prescribed ruxolitinib 1.5% cream to be applied twice daily; we also discontinued the metronidazole and doxycycline. At his follow-up visit only 2 weeks later, he had achieved complete resolution of the SD (Fig 1), with a partial decrease of the erythema of rosacea.

## Discussion

SD is a chronic and common inflammatory skin disease associated with reduced quality of life. When first-line treatments fail, additional therapies are necessary to manage symptoms and prevent recurrences. Topical ruxolitinib cream was approved by the Food and Drug Administration in late 2021 for the short-term and noncontinuous treatment of mild-to-moderate atopic dermatitis in nonimmunocompromised patients aged 12 and up. Ruxolitinib is a Janus kinase (JAK) inhibitor, which selectively targets JAK1 and JAK2.^7^ In atopic dermatitis, inflammation is driven by type 2 cytokines, which are modulated by JAKs.^7^ In clinical trials, patients with AD experienced rapid and sustained skin improvement with the use of topical ruxolitinib, which provided potent antipruritic and anti-inflammatory effects.^7^ Similarly, due to reports (albeit preliminary) that the inflammation in SD is driven by interleukin 4 and interleukin 17,^4^ similar mechanisms of action could explain the effect of ruxolitinib in ameliorating both spongiotic diseases. Topical ruxolitinib has also been reported as an effective new therapy for vitiligo and lichen planus—diseases driven by elevated levels of interferon gamma, an upstream effector of JAKs.^8^^,^^9^ In these reports, ruxolitinib 1.5% cream provided significant repigmentation^8^ and rapid improvement^9^ of facial vitiligo and cutaneous lichen planus, respectively. To our knowledge, this is the first report describing the excellent efficacy of ruxolitinib in a patient with significant SD and concomitant rosacea. This case highlights the promising role of topical ruxolitinib cream in the treatment of facial SD.

## Conflicts of interest

None disclosed.
